# Identification of papilloedema in general practice in England: a mixed-methods cross-sectional study

**DOI:** 10.1136/bmjopen-2026-121321

**Published:** 2026-10-29

**Authors:** Olivia A Skrobot, Jonathan AK Chin, Matthew J Ridd, Samuel W D Merriel, Denize Atan, Alyson L Huntley

**Affiliations:** 1 Centre for Academic Primary Care, Bristol University, Bristol, UK; 2 Bristol Medical School, Bristol, UK; 3 Centre for Primary Care and Health Services Research, The University of Manchester, Manchester, UK

**Keywords:** OPHTHALMOLOGY, Primary Health Care, Health Services Accessibility, Surveys and Questionnaires

## Abstract

**Abstract:**

**Background:**

Papilloedema, defined as optic nerve swelling caused by raised intracranial pressure, may present with headache and can indicate serious underlying intracranial disease. General practitioners (GPs) encounter papilloedema infrequently yet missed diagnoses may result in delayed identification of potentially life-threatening conditions. Recognising papilloedema in primary care is often challenging.

**Objective:**

To explore GPs’ perspectives on identifying and referring patients with papilloedema.

**Design, setting and participants:**

A mixed-methods cross-sectional study of GPs in England.

**Intervention/methods:**

Six National Institute for Health and Care Research Clinical Research Networks disseminated a questionnaire to GPs (June to October 2023); responses were analysed using quantitative/qualitative methods. Five GPs who completed the questionnaire were interviewed by video call. Interviews were thematically analysed.

**Results:**

45 GPs completed the questionnaire. In a case vignette presenting with headache, most GPs (78%) would perform fundoscopy, but were not confident in identifying papilloedema. Nearly half (49%) recommended the case to see a community optometrist who was considered better skilled and equipped to examine eyes. GPs said current practices could be improved by allowing optometrists to refer patients directly to appropriate hospital specialists.

Four themes were identified from five interviews: the GP’s role in suspecting papilloedema; challenges in assessing patients for papilloedema; the impact when local protocols and pathways for suspected papilloedema are not available; and the need for clarity and support in dealing with suspected papilloedema.

**Conclusion:**

Although GPs rarely encounter papilloedema, delayed or missed diagnosis may result in serious morbidity. The development of clear clinical pathways for assessing patients presenting with headache and optic disc swelling in primary care could support earlier recognition and appropriate referral.

**STRENGTHS AND LIMITATIONS OF THIS STUDY**

- The study used mixed methods, consisting of a questionnaire, follow-up interviews and established analysis methods, to gain a more detailed understanding of the topic.
- Academic general practitioners (GPs) practising in England helped to write the questions and with the piloting of the questionnaire.
- While six clinical research networks in England agreed to disseminate the invitation to practising GPs, these networks did not cover the North of England.
- The survey and interview sample sizes were modest; however, within the context of the broader study, there were no obvious indications of bias.

## Introduction

 Papilloedema refers to optic nerve swelling caused by raised intracranial pressure (ICP). Papilloedema is an important clinical sign to pick up in community settings as it is a headache red flag that could indicate underlying life-threatening disease, for example, brain tumours.^1 2^ Patients with papilloedema need urgent hospital investigations to determine the cause. However, headaches are common and the positive predictive value of headache is only 0.2% for a brain tumour without other symptoms or signs.^3^

Fundoscopy is frequently performed on patients with headache to look for signs of papilloedema but in some patients, normal variation of their optic nerve anatomy can be mistaken for swelling, known as pseudopapilloedema. Even health professionals trained to recognise eye abnormalities, such as optometrists working in community practices, can struggle to distinguish papilloedema from pseudopapilloedema and benign headache symptoms from secondary causes. See table 1 for glossary of terms.

**Table 1:** Glossary of terms

Term	Definition/description
Papilloedema	Optic nerve swelling that is specifically caused by raised intracranial pressure.
Optic disc swelling	Swelling of the anterior end of the optic nerve within the eye that can be visualised by fundoscopy; it can be caused by many different aetiologies, and most are unrelated to intracranial pressure, eg, ischaemia, inflammation, nutritional deficiencies, inherited gene mutations. Swelling causes elevation of optic disc.
Pseudopapilloedema	An umbrella term that refers to any benign variant in optic disc appearance that can be mistaken for optic disc swelling or papilloedema, generally because the disc(s) appears to be elevated. However, swelling is not truly present.
Crowded optic disc	A variant of normal optic disc anatomy, in which a normal number of nerve fibres are squeezed through a smaller than average opening in the sclera, leading to an elevated disc profile that can be mistaken for optic disc swelling/papilloedema. It is more common in people who are long-sighted.
Optic disc drusen	These are protein deposits; initially buried within the fibres of the optic nerve head, they become calcified over time and migrate to the surface of the optic disc when they become visible by fundoscopy. While they are buried and hidden within the nerve fibres of the optic disc head, they can be mistaken for optic disc swelling/papilloedema because they elevate the disc’s profile (as with other causes of pseudopapilloedema).

Due to diagnostic uncertainty, and the potentially serious consequences of missing papilloedema, many people are referred from the community to emergency and specialist hospital services for suspected papilloedema when alternative approaches could have been taken.^4^ The conviction of an optometrist in England for gross negligence manslaughter after they failed to identify papilloedema in a child who later died has made optometrists more anxious about missing the diagnosis.^5^ As a result, hospital eye services across the UK have reported large numbers of optometrist referrals of people with suspected papilloedema who have turned out to have primary headache or pseudopapilloedema and could have been managed in the community.^6–8^ Furthermore, increased demand on hospital eye services can result in the delayed diagnosis and treatment of people with serious health problems, like brain tumours, leading to worse health outcomes.^9^

This study aimed to explore general practitioners (GPs)’ perspectives on identifying and referring patients with papilloedema. We focused on factors that influenced their decisions to refer, and the availability of local guidance and referral pathways for patients with suspected papilloedema. It is part of a larger study that has also surveyed optometrists, ophthalmologists, neurologists and emergency care clinicians and with additional interviews with optometrists and ophthalmologists about their perspectives on this topic.

## Methods

### Study design

A mixed-methods cross-sectional study of GPs in England. The study surveyed GPs with an online questionnaire to capture an overview of the research aim cited above; topics that arose from the surveys were explored in greater depth in semi-structured interviews.

### Population

From June to October 2023, 6 of the 15 National Institute for Health and Care Research Clinical Research Networks (CRNs) (West of England, Southwest Peninsula, East Midlands, West Midlands, South London, Kent, Surrey and Sussex) disseminated study invitations to research active general practices. Because the number of recipients was not recorded, a survey response rate could not be determined. Participants completed electronic informed consent before the questionnaire, with optional consent for a follow-up interview.

### Questionnaire

The questionnaire was based around two case vignettes. Vignette 1 presented with headache and a colour fundal photo of pseudopapilloedema; Vignette 2 was an optometrist referral letter about an asymptomatic patient with ‘raised, blurred, and indistinct’ optic disc margins, a description that does not discriminate between papilloedema or pseudopapilloedema. The case vignettes were based on the clinical presentations of real-world patients referred to a UK-based hospital eye service (DA), which typically create the greatest diagnostic uncertainty. The fundal photograph was used with the informed consent of the patient.

The survey questions explored how GPs would take a more detailed clinical history, examine and manage the patients. The availability of local referral protocols and perceived trends in referrals were also surveyed. Additional questions were created with our patient and public involvement and engagement (PPIE) group to ask what information GPs should provide to patients about the differential diagnosis and what to expect if referred to hospital.

The DIagnostic accuracy of referral for PaPilloedema (DIPP) surveys were initially developed by the neuro-ophthalmologist (DA) and health services researchers (AH and OS) within the DIPP study team. The surveys were subsequently tailored to each professional group within the referral pathway following consultation with representatives from the relevant disciplines. Feedback was obtained through meetings and email correspondence with at least one health professional from each target group, including GPs, optometrists, emergency care clinicians, ophthalmologists and neurologists.

The GP survey was piloted by MJR and SWDM, who were invited to join the DIPP study team specifically to provide general practice perspectives and expertise. Following pilot testing, several modifications were made to improve the clarity, relevance and accessibility of the survey for GPs. For example, the question in case 1 asking, ‘What would you ask about in the clinical history?’ was changed from free-text response to a multiple-choice format. In addition, a named legal case included in a question exploring factors influencing referral decisions was replaced with the more general description, ‘Litigation case against an optometrist for missing papilloedema’.

### Semi-structured interviews

GPs were selected by purposive sampling, to ensure there was representation of GPs from different parts of England.^10^ Interviews were conducted online for up to 30 min by a female senior research associate (OS), trained in qualitative research methods and using a bespoke topic guide designed by the study team to provide context to the semi-quantitative data of the survey by exploring real life experiences (online supplemental A1).

### Analysis

Only fully completed questionnaires were included in the analysis. Summary statistics of questionnaire data and their graphical representation were produced in Microsoft Excel. Narrative summaries of free-text responses were collated. Integration of these data were convergent and is presented within the framework of the two case vignettes from the survey.

Thematic analysis of the interviews, based on Braun and Clarke methodology, was conducted by two researchers (OS and AH) and a final year medical student (JAC) who independently coded in Microsoft Excel.^11^ Discussions were held to formulate consensus on the codes, categories and the overarching themes that were derived from the data (online supplemental A2). Some of these themes were also apparent in the responses to the questionnaire and so supportive findings in the form of statements and quantitative data from the questionnaires are presented within these themes.

We met with our PPIE groups prior to completing this paper for comments and discussion for the next steps of the DIPP study.

### Reporting

We have used the Strengthening the Reporting of Observational Studies in Epidemiology (STROBE) checklist of items for cross-sectional studies and the Good Reporting of a Mixed Methods Study (GRAMMS) criterion for mixed method research to guide our reporting of this study (online supplemental checklists).^12 13^

### Patient and public involvement and engagement group

The DIPP study has two PPIE groups: a hospital PPIE group (six patients with brain tumours) and a community PPIE group (six members of the public who use optician services). The two groups chose to remain separate to allow full uninhibited expression of their opinions. We have endeavoured to get a representative mixture of ages, ethnicity and gender. Contact with our PPIE groups is varied based on the contributors’ preferences.

## Results

### Participants

45 GPs completed the questionnaire; the majority were GP partners (71%) and had over 10 years’ experience (69%) (online supplemental B1). Four GPs started to answer the questionnaire but did not attempt any further than case vignette 1 and were not included in the analysis. Five GPs from different regions of England were interviewed.

### Questionnaire findings

#### Vignette 1: (45 respondents)

All participants said they would ask the patient about neurological symptoms and the factors that exacerbated or eased their headaches (online supplemental B2). Nearly all, 96% of GP respondents, would measure blood pressure; 84% would examine the cranial nerves; and 61% would examine their limbs neurologically (online supplemental file 1).

Just under half, 47% of GPs, mistook the image for papilloedema but only 24% were confident in their assessment. Only 1/3 GPs who had correctly identified the patient to have pseudopapilloedema was confident about their assessment; and 29% of GPs said they did not know what the image showed (online supplemental B2).

GP respondents varied in their management of the patient: 49% recommended the patient to see a community optometrist rather than referring them to hospital (figure 1), irrespective of whether they thought the patient had papilloedema or not.

I would like someone who has the appropriate equipment and experience in examining the fundus to look. In the real world I will not have been able to get such a good view of the fundus as in the photograph so my confidence at diagnosis would be even lower. I would hope they could be examined swiftly in the community - if this was not possible, I would refer urgently to ophthalmologist for them to examine.

**Figure 1:**
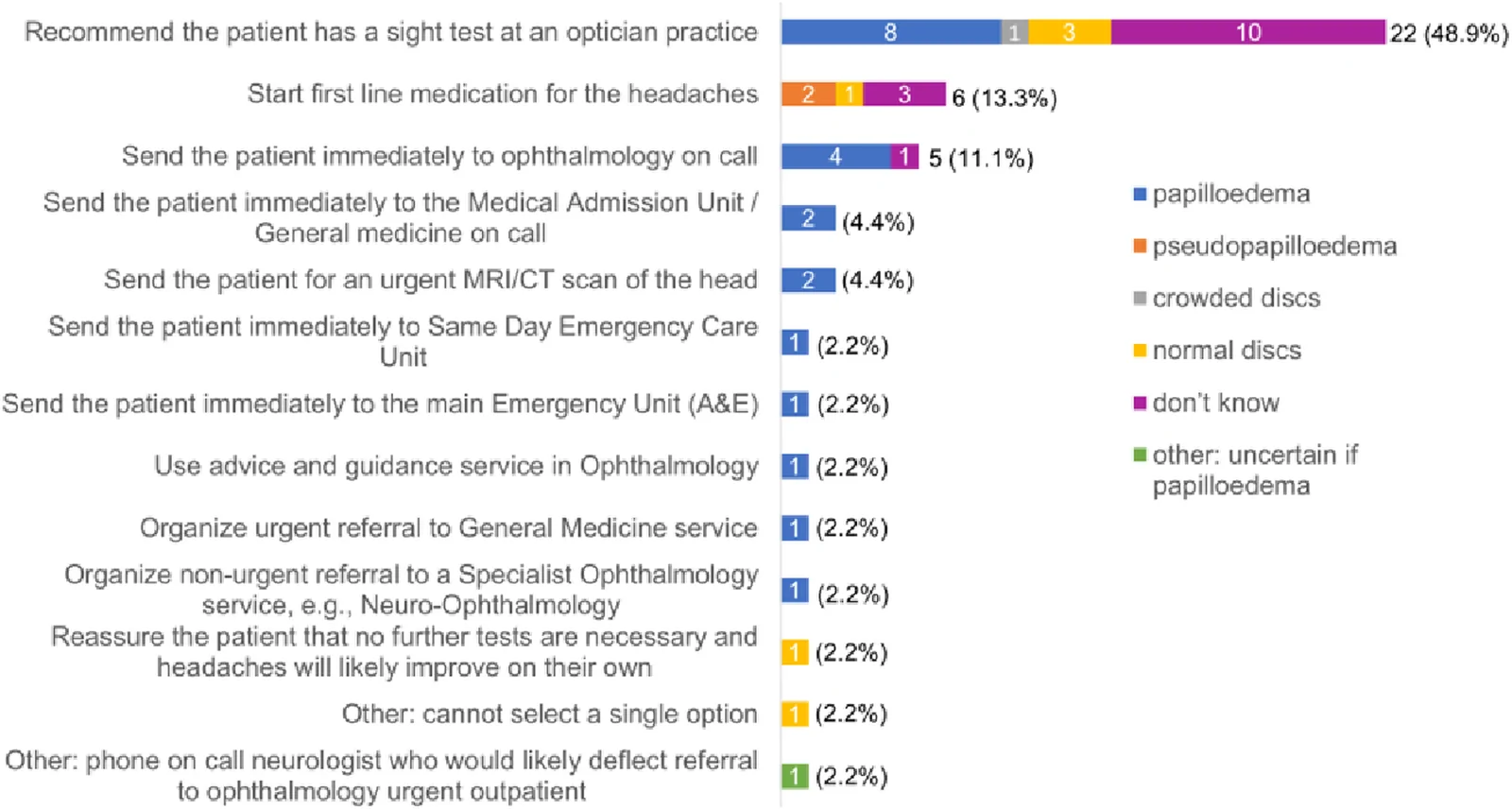
GPs varied in their management of case 1 based on their assessment of the patient’s disc appearances. Accident and Emergency; GPs, general practitioners.

#### Vignette 2: (45 respondents)

The management of this case differed between GPs; 45% felt they could not make a referral decision without first examining the patient, and 13% felt they needed to speak with the patient to gain further information before determining what to do (online supplemental B3) (figure 2).

**Figure 2:**
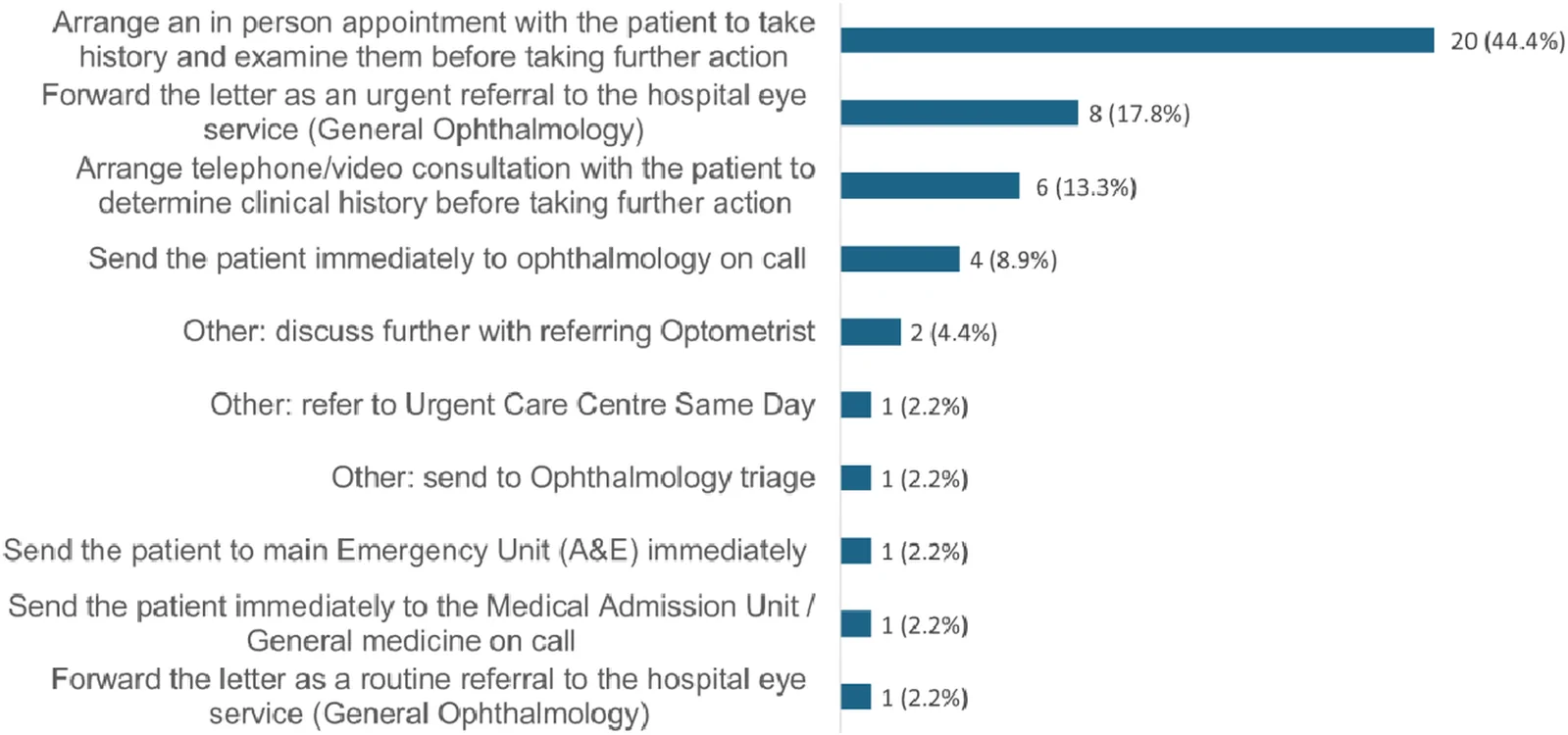
Most GPs would arrange a consultation with case 2 before deciding whether hospital referral was appropriate. A&E, Accident and Emergency; GPs, general practitioners.

The case highlighted regional differences in the ability of optometrists to directly refer patients to secondary care. Some GPs questioned why the optometrist had written to them as this was not the pathway in their region. One GP commented, ‘This still occurs, why are opticians not referring directly rather than everything going via general practice as an intermediary?’*,* whereas another GP stated they would rely on the recommendation of the optometrist on how to manage the patient.

GPs said they would refer the patient to different hospital departments based on whether they thought it was necessary to determine whether papilloedema was present (eg, via ophthalmology), or to arrange urgent investigation (eg, with brain imaging), considering the fastest/most accessible route for the patient.

“[urgent referral to General Ophthalmology is] a fast way for the patient to be seen. As he is well I wouldn’t want him to sit in A&E for 8 hours before being seen by a junior doctor*.*”

We developed questions around referral communication (online supplemental B3) with our PPIE groups. We asked GPs which information they thought would provide the greatest reassurance to patients that they were receiving appropriate care when the GP was either certain or uncertain about their identification of papilloedema. When GPs were certain of their identification of papilloedema, 87% responded that discussing what to expect following hospital referral would reassure patients, 73% felt that explaining the range of possible underlying diagnoses would be reassuring, and 60% thought that discussing expected waiting times for appointments would be helpful. When GPs were uncertain of their identification of papilloedema, 82% responded that discussing what to expect following referral would reassure patients and 80% thought that explaining the differential diagnosis would be reassuring. Fewer GPs (44%) considered discussion of appointment waiting times to be reassuring in the context of diagnostic uncertainty. GPs appeared to think that leaflets and online information were less helpful (online supplemental B3).

Other findings from the questionnaire are presented within the following section, as well as in online supplemental B3: referral trends.

### Thematic analysis of interviews

Our thematic synthesis of the GP interviews produced 21 codes that fitted into eight categories to form four final themes and five subthemes. Some codes fed into more than one category, and more than one category fitted into four main themes. The main relationships are shown in figure 3.

**Figure 3:**
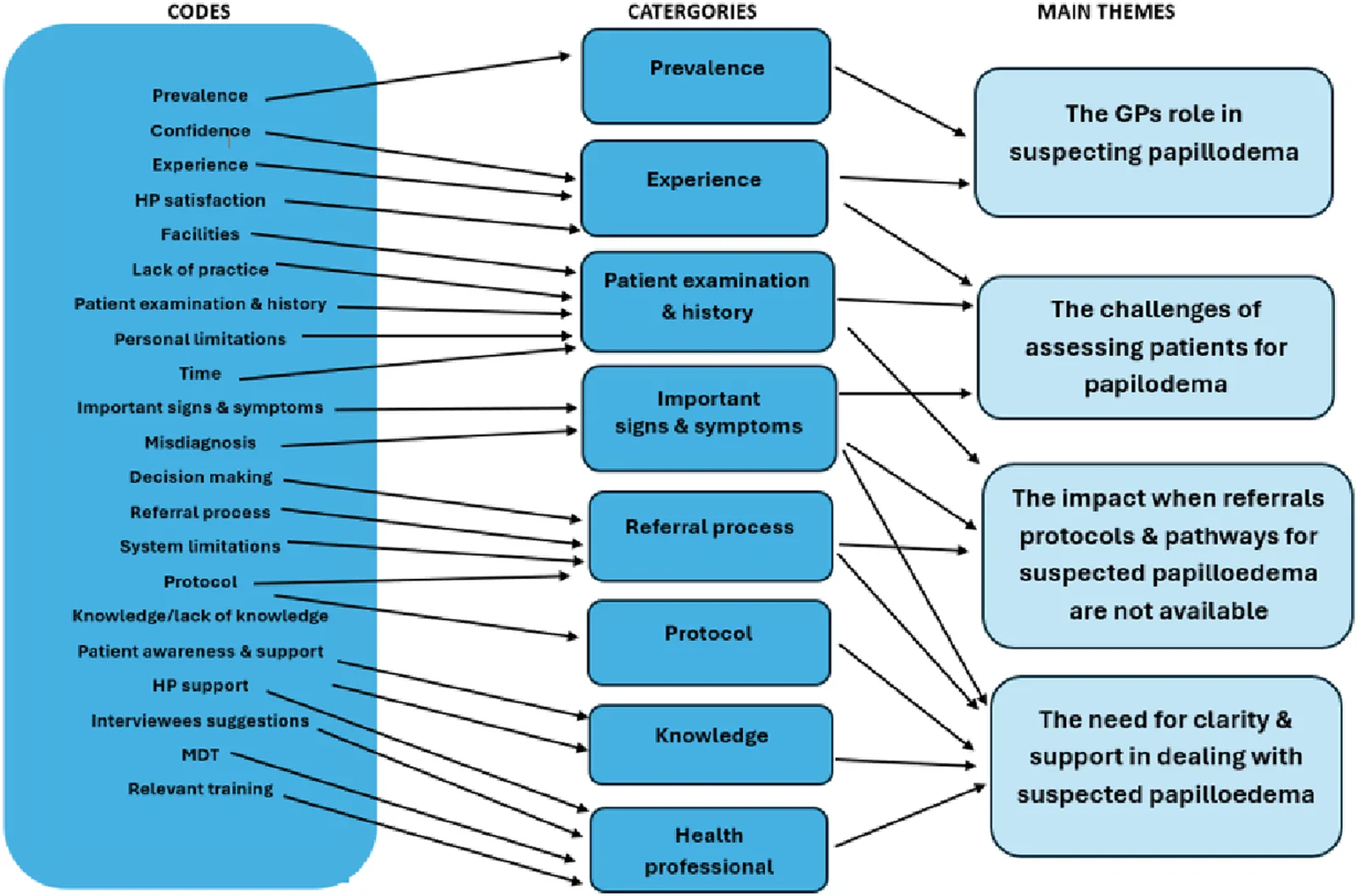
Thematic synthesis of interview data. GP, general practitioner; HP, health professional; MDT, multidisciplinary team.

The four final themes were: the GP’s role in suspecting papilloedema, the challenges of assessing patients for papilloedema, the impact when referral protocols and pathways for suspected papilloedema are not available and the need for clarity and support in dealing with suspected papilloedema. The supporting illustrative quotes can be found in table 2. These themes were also apparent in the responses to the questionnaire. There were some cross-cutting elements across the themes, such as GP time and clinical responsibility.

**Table 2:** Illustrative quotes from semi-structured interviews

Theme	Illustrative quotes
The GP’s role in suspecting papilloedema	**A1:** “So obviously, I think I would still recognize it. But it’s not something I’m regularly seeing or assessing I’m not doing fundoscopy very often, so I wouldn’t feel that confident.” (GP 1) **A2:** “I haven’t seen it much. And I probably don’t look at the back of eyes often enough, either… It’s not something you’re doing every day as a GP probably not even something you’re doing every month as a GP.” (GP 5) **A3:** “So, if their blood pressure is very high in patients with neurological symptoms and signs, particularly headaches, …they have any red flag, or concerning symptoms, I might take a look at the back of their eye. Also, sometimes in other kind of symptoms where I’m concerned about a neurological condition, I might look at the back of their eyes as well, in that situation.” (GP 4)
The challenges of assessing patients for papilloedema	**B1:** “Medical students do…some ophthalmology as part of their undergraduate teaching. But it’s pretty brief to be honest. So, you learn how to use an ophthalmoscope. Things to look out for at the back of the eye when you’re doing that training…there’s not a huge amount of training on ophthalmology after that in general practice.” (GP 4) **B2:** “When I started out medicine, I used to use ophthalmoscope a lot, but as time’s gone on, I’ve used it less and less. So. It’s the skill that you fall out of practice with.”…“So, it’s lack of practice…because it’s a relatively rare diagnosis.” (GP 2) **B3:** “We don’t necessarily have the time to dilate pupils and darken room and all the things that you should do to do a proper examination, get a really good look at the back of someone’s eyes.” (GP 4) **B4:** “Yeah, I think the biggest problem we have is time. …In an ideal world we would dilate people’s eyes up and have a good look and be more experienced. But that doesn’t happen. And then I don’t think it is gonna happen in general practice. I think if we got concerns they should be seen elsewhere.” (GP 3)
The impact when referral protocols and pathways for suspected papilloedema are not available	**C1:** “There’s a form that they fill asking us to make referral with their findings on. But I wouldn't see the patients. I would just add in sort of past medical history, drug history, etc. and forward it onto ophthalmology if they ask me to.” (GP 1) **C2:** “I guess that the one that always is a bit more tricky are the ones we get from opticians where …opticians write to us, saying, Oh, this patient’s got papilloedema, please refer. And without knowing what clinically, what’s going on the patient, it’s very difficult to know quite what the optician is worried about or concerned about or what, how urgent the problem is in that situation….they won’t necessarily give us a huge amount of other the clinical information. So normally…I would want to speak to the patient myself in that situation to find out what kind of symptoms or problems that they have.” (GP 4) **C3:** “it could affect things, it might make you less likely to try to refer to a neurologist. And you might think I’ll refer ophthalmology first cause you just kind of anticipate a rejection. And then it’s more work and difficulty for the patient.” (GP 1) **C4:** “If I refer on the choose and book on the routine process. Patients don’t get seen for 3 to 6 months, so I wouldn’t feel comfortable doing that. So, I tend to always send to A&E.” (GP 2) **C5:** “this is always one of the challenges. I’ve had patients where … opticians have said, can you please refer to ophthalmology to be seen within 1 to 2 weeks, and that when I've done it patients have been waiting far, far, far, far longer than 1–2 weeks.” (GP 5)
The need for clarity and support in dealing with suspected papilloedema	**D1:** “I think it’s a bit of both. I’d like to improve my confidence, but I’d also like there to be good pathways so that I’m not making that judgment call necessarily entirely on my own.” (GP 5) **D2:** “it always feels a little bit awkward because sometimes routine ophthalmology referrals can sometimes take a long time. So there is always that slight concern…if there is something more concerning going on… is that good that they’re gonna be waiting for too long before they get seen?” (GP 4) **D3:** “we actually have optometrists embedded into our referral service, so they will actually triage the referrals for us… and they then decide on where the best place for that person is to be seen, and what urgency and that type of thing.” (GP 4) **D4:** “it would give greater clarity for GPs about when they should be concerned if they do suspect papilloedema, or what actions they should take if they do find it, particularly if they find it in someone who doesn’t have symptoms, what level of concerns they should have where they should refer patients at that point. So, I think, guidelines that that are useful for both opticians and doctors.” (GP 4)

A&E, Accident and Emergency; GP, general practitioner.

### The GP’s role in suspecting papilloedema

When asked about examining patients for papilloedema, GPs expressed two main concerns. First, that they had relatively limited experience of the clinical scenario (table 2A1 and A2). Second, GPs said that identifying papilloedema is not a major part of their role, and they tend to consider the symptoms and signs of a patient in a more holistic way suspecting a patient might have papilloedema may be part of that (table 2 A3).

When asked whether they thought hospital referrals from GPs and optometrists had changed over the last 5–10 years for suspected papilloedema, >50% responded that they had not noticed any changes, although nearly a quarter stated they did not know (online supplemental B3).

### The challenges of assessing patients for papilloedema

GPs described a range of barriers that impaired their ability to examine the eyes of a patient they suspect to have papilloedema.

Subtheme - lack of training: GPs have minimal training and educational opportunities to perform fundoscopy beyond their undergraduate years (table 2B1). Despite good training at the beginning of their career, one GP felt that they became rapidly deskilled in fundoscopy due to lack of ongoing support/training and limited exposure to cases of suspected papilloedema (table 2B2).

Subtheme - lack of time and other practical barriers: GPs stressed that lack of time was a significant limiting factor to their ability to examine the eyes of a patient, along with equipment availability and difficulties in adjusting the room lighting (tables 2B3 and 4). As a result, GPs who suspect a patient might have papilloedema from their preliminary assessment would look to refer the patient to an optometrist or hospital.

### The impact when referral protocols and pathways for suspected papilloedema are not available

Subtheme - difficulty knowing how to act when optometrists suspect papilloedema: Responses to the questionnaire and interviews showed that working relationships and communication between optometrists and GPs vary significantly. There is also wide variation in referral pathways between primary and secondary care in different regions of England.

The five GPs interviewed had mixed experiences of, and opinions about how they should act on letters from optometrists. Two GPs had received optometrist letters and had different approaches. The first GP was comfortable in trusting the clinical judgement of the optometrist about whether referral to secondary care was warranted and the degree of urgency required. The second GP wanted to see patients to understand the clinical concerns before deciding how to act (table 2C1 and C2). Of the remaining three GPs, two had not received referral letters from optometrists about suspected papilloedema, and the third GP speculated that community optometrists in their region had the power to make urgent referrals directly to secondary care.

Subtheme - lack of available local protocols: One of the main concerns expressed by GPs was the lack of a clear referral pathway to secondary care for suspected papilloedema. Over half (58%) of GP respondents to the questionnaire referred to guidelines published by professional bodies, for example, Royal College of General Practitioners, and National Institute for Health and Care Excellence clinical knowledge summaries but few GPs referred to local protocols. Around 40% of GPs said they did not know any local protocols for suspected papilloedema (figure 4). As a result, most GPs surveyed could not provide an opinion on whether local referral pathways for patients with suspected papilloedema worked well in their region (47% responded ‘don’t know’; 16% responded ‘not applicable’). Of the GPs who were aware of local protocols: 16% said they worked but could be improved; 13% said they work very well; 9% said they are unclear and should be simplified. Their choice of where to refer, however, could be influenced by perceived barriers to referral and communication that were local to their region (table 2C3).

**Figure 4:**
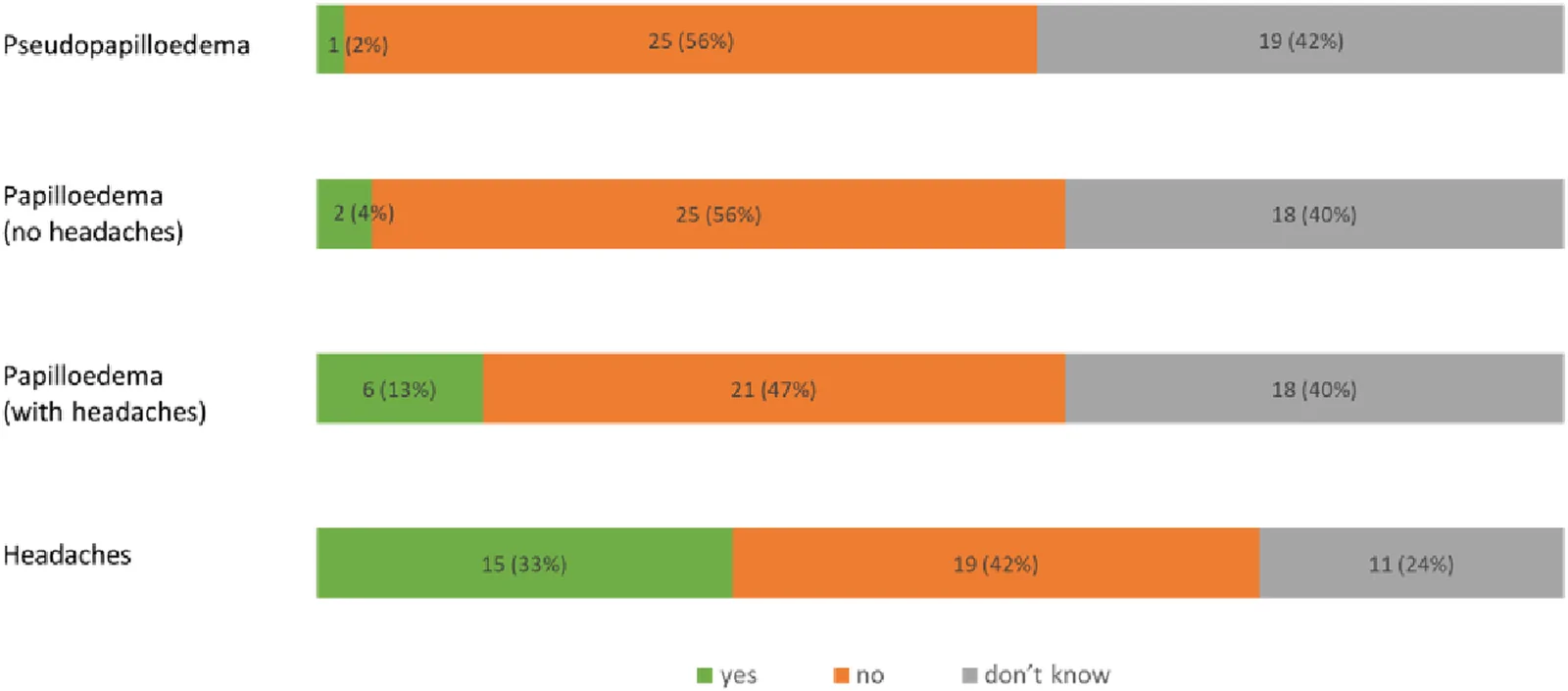
There is variation in the availability of local referral protocols to help decide when and who to refer patients with pseudopapilloedema, papilloedema and/or headache to in primary or secondary care.

Subtheme - uncertainty about required urgency of referrals for suspected papilloedema: GP decision-making was influenced by the time it would take for a patient to be seen if they were referred to an outpatient service rather than an emergency department. GPs stated that the lack of a clear pathway, alongside their clinical uncertainty in identifying papilloedema, often resulted in their sending patients to the emergency department, potentially inappropriately (tables 2C4 and 5).

If concerned about waiting times for hospital appointments for suspected papilloedema, 60% of GPs answering the questionnaire would emphasise headache red flags in their referral letter/email, 53% would call the ophthalmology department to discuss the patient, 33% suggested the patient should be seen urgently/within a certain time frame in their referral letter/email; and 27% would tell the patient to go to Accident and Emergency (A&E (online supplemental B3) (table 2C4).

Other GPs were comfortable with the referral options available to them, depending on how certain they were that the patient had papilloedema (table 2 C5). The lack of availability of clear referral protocols and pathways is likely to have contributed to the range in responses by GPs to the management of the case vignettes.

### GP’s need for clarity and support in dealing with suspected papilloedema

Interviewees welcomed further training and support in both the clinical assessment and management of such patients. GPs wanted more clarity on what clinical situations they should be able to consider as a less urgent referral, for example, for pseudopapilloedema (table 2 D1 and D2).

GPs spoke of valuing optometrists’ opinions, suggesting a close working relationship would be beneficial for dealing with suspected papilloedema or pseudopapilloedema. GPs thought guidelines for papilloedema and pseudopapilloedema would be helpful (table 2 D3 and D4).

Several GPs suggested direct referral from optometrists to specialist services would help improve current practice. One of the interviewees suggested a pathway through optometrists, like the minor eye condition scheme commissioned in many regions of England, would help to limit diagnostic delay. Some GPs stated referral to the eye casualty worked well. Another GP suggested it would be useful to make doctors aware of wait times and include a checklist of symptoms on the referral form to improve triage.

## Discussion

People with papilloedema from raised ICP rarely present to GPs, and it is an uncommon reason for them to refer patients to secondary care. Despite the rise in referrals for suspected papilloedema reported by hospital eye services in the UK,^5^ most GPs had not appreciated any changes in referral rates from optometrists over the last 5–10 years, although some said optometrists could refer patients directly to hospital in their area and nearly a quarter were uncertain about referral trends. Most GPs said they perform fundoscopy as part of their cranial nerve examination of patients with headache to look for papilloedema. However, GPs expressed their lack of confidence and practice in examining the eye for papilloedema; further citing lack of available equipment or suitable environment for eye examinations, time and basic training. GPs in our study expressed a need for clearer guidance on the appropriate use of community optometry services and referral pathways to secondary care for patients presenting with headache and suspected papilloedema. Such guidance may support more consistent decision-making, facilitate timely identification of patients with raised ICP and optimise the use of healthcare resources. However, further research is required to evaluate the clinical effectiveness and cost-effectiveness of implementing these pathways, including their impact on diagnostic timeliness, patient outcomes, referral patterns and National Health Service (NHS) resource utilisation.

### Strengths and limitations

A strength of our mixed method approach was that respondents to the questionnaire had the opportunity to provide free-text answers to explain their responses to closed questions and provided greater insight into their decisions and management of the case vignettes. Many of the topics that arose from the questionnaire were discussed in depth during the interviews with GPs. This approach facilitated the integration of both the quantitative and qualitative questionnaire data into the themes generated from the interviews.

CRNs were an effective means to disseminate our questionnaire to GPs; however, none participated from the north of England and therefore recruitment from general practice from this region was not possible. We acknowledge that the number of GPs participating in the survey and interviews was modest and limited to members of the CRN. As referral pathways for suspected papilloedema vary across the UK, it is possible that some perspectives were not captured, which may limit the generalisability of the findings.

However, this work should be considered within the broader context of the DIPP study, which also included surveys and interviews with optometrists and ophthalmologists regarding the same referral pathway.^14^ Although these findings have been reported separately for practical reasons, participants from different professional groups frequently commented on the roles, experiences and challenges faced by others within the pathway. The consistency of these observations with the views expressed by GPs provides reassurance that no major sources of bias were evident. In addition, the interview data were broadly consistent with the survey findings and provided valuable contextual insights that helped explain and confirm the quantitative results. Taken together, we believe that the participants provided a sufficiently diverse range of perspectives to address the study objectives. While a larger survey and additional interviews may have identified further nuances, we consider it unlikely that they would have materially altered the principal findings, particularly given the practical constraints of finite research resources.

A further limitation was that our study name may have introduced bias. The DIPP study ‘Improving the DIagnostic accuracy of referrals for PaPilloedema’ implies that most referrals currently are inaccurate; however, in the questionnaire and interviews, participants were not asked to comment on the accuracy of referrals in their region.

Currently, community optometry provision is limited by the General Ophthalmic Services contract in England, which only provides NHS funding to optometrists to perform sight tests for spectacles with no additional investigations such as fundal imaging that would be helpful in distinguishing papilloedema from pseudopapilloedema. Additional NHS funding is available for enhanced optometric services, for example, for glaucoma and cataract screening or minor eye care services in the community, but their provision is patchy in different regions of England, which means many patients are referred to hospital for further investigation when some tests could be performed in the community.^15^ A new model ‘Optometry First’ developed to provide a more collaborative primary eye care service and to alleviate the burden on hospital eye services was proposed in 2022 and is being piloted in three areas in England, which may address some of these issues.^16 17^

The aim of this work is to gather data to inform a modified Delphi process to reach consensus among experienced and expert stakeholders on clinical guidelines that will help support health professionals to accurately identify people with papilloedema from pseudopapilloedema and headache symptoms from primary versus secondary causes in primary care and manage them as appropriate. The DIPP guidelines will be trialled, evaluated and subsequently proposed for adoption in England.

## Conclusion

GPs reported limited exposure to, and confidence in identifying headache patients with papilloedema from raised ICP. Participants highlighted the need for additional training, and sufficient consultation time to assess for papilloedema, an important headache red flag. GPs supported the development of clear clinical guidance to aid: (1) recognition of headache presentations and signs of papilloedema from raised ICP requiring urgent hospital referral; (2) appropriate referral to community optometrists and interpretation of ophthalmic findings; and (3) management of patients with benign headache disorders or pseudopapilloedema within the community. Further research is needed to determine whether implementation of such guidance could reduce diagnostic delays for patients with headache and papilloedema due to raised ICP, while improving referral pathways and reducing unnecessary healthcare utilisation and costs.

## Supplementary material

10.1136/bmjopen-2026-121321 online supplemental file 1

10.1136/bmjopen-2026-121321 online supplemental file 2

## Data Availability

Data are available upon reasonable request.
