# Supplementary material for: Identification of papilloedema in general practice in England: a mixed-methods cross-sectional study
Source: BMJ Open. 2026 Oct 29;16(10):e121321. doi: 10.1136/bmjopen-2026-121321 (PMC13723832; doi:10.1136/bmjopen-2026-121321)
Supplement: Supplementary file 1 [file bmjopen-16-10-s001.pdf]

Supplementary materials

A) Supplementary methods

A1. Semi-structured interview guide

Diagnosis and decision-making

How often would you see a patient with suspected papilloedema
Can you talk me through a time in which you have assessed a patient for papilloedema?
How confident did you/would you feel in assessing them?
Prompt: Do you think you have the appropriate equipment or time to assess them?
[if appropriate] Would you like to be more confident?
What factors influenced / would influence your decision on how to manage that patient?
Prompt: Do you have a protocol or guidelines to help you decide how to manage these case scenarios with suspected papilloedema?
Prompt: What would help you decide the urgency of referral?
Prompt: What options do you have if you are unsure if the patient needs to go to secondary care, but you can't make a judgement on your assessment.

Patient information

Can you describe the conversation you had with the patient / in that scenario [when the diagnosis is uncertain]?
Would you /did you provide any information sheets or online resources to your patients?

Referral process

Do you receive referrals from opticians for patients with suspected papilloedema?
Do you always accept the referrals or is there a threshold/certain level of information required to accept them?
Does secondary care always accept the referrals?

Opinion of referrals

Has the number of referrals from opticians you receive changed in the last 5-10 years?
Do you think the number of patients that you have seen with papilloedema has changed in that time?

Barriers and facilitators

Is a need to change the management of patients with suspected papilloedema?
Do you think the guidelines for managing patients with papilloedema that we aim to develop would be useful for GPs?

Prompt: what would be most useful to help you?

Is there anything else you feel important to talk about regarding this topic?

A2. Process of thematic analysis of interview transcripts.

Coding

- Two researchers independently coded each transcript
- Codes were compared and discussions held with a third researcher to agree final coding
- 21 codes were agreed: Confidence, Decision Making, Experience, Facilities, Health Professional Satisfaction, Health Professional Support, Important Signs and Symptoms, Interviewee Suggestions, Knowledge/Lack of Knowledge, Lack of Practice, MDT, Misdiagnosis, Patient Awareness and Support, Patient History and Examination, Personal Limitations, Prevalence, Protocol, Referral Process, Relevant Training, System Limitations, Time
- Coded transcript from all interviews were compiled
- Codes were summarised

Categories

- Two researchers independently developed categories to encompass the codes
- Categories were compared and discussions held with a third researcher to agree 8 final categories: Experience; Patient examination and history; Important signs and symptoms; Referral process; Prevalence; Knowledge; Protocol; and Health Professional support.

Themes

- Three researchers independently developed overarching themes
- Themes were compared and discussions held to develop final themes
- One researcher wrote the final themes with representative quotes
- All three researchers reviewed and agreed the final 4 themes

B) Supplementary results from questionnaire

B1 Participant characteristics

What is your current grade/job title?	***How long have you been working in General Practice?***					
	0-2 years	***2-5 years***	***5-10 years***	***10-20 years***	***20+ years***	***Total***
GP Trainee	*1*	*0*	*0*	*0*	*0*	*1 (2%)*
Locum GP	*0*	*1*	*0*	*0*	*1*	*2 (4%)*
Salaried GP	*1*	*2*	*1*	*4*	*2*	*10 (22%)*
GP partner	*1*	*2*	*5*	*14*	*10*	*32 (71%)*
Total	*3 (6.7%)*	*5 (11.1%)*	*6 (13.3%)*	*18 (40%)*	*13 (28.9%)*	*45*

Table 1: Types of General Practitioner (GP) and their number of years working in general practice.

B2 Case vignette 1: additional results and figures

Case 1: A woman in her twenties presents to your practice with a history of headaches for the last 4 months. She is overweight with a BMI over 30. She has no past medical history and does not take any medication or analgesia for her headaches.

Q: What additional questions about the clinical history would you ask?

All GP respondents would ask case 1 about neurological symptoms and the factors that exacerbated or reduced their headaches. All but 1 GP (44/45, 98%) would ask about problems with vision; nausea/vomiting; medications; and duration and frequency of the headaches. The majority (40/45, 89%) would ask about photosensitivity (figure 1).

Five GPs asked about additional lines of enquiry: 4/5 about psychological risk factors (stress, anxiety, current life events, social history); 2/5 about timing of headache in relation to premenstrual tension/menstrual cycle; 2/5 about diurnal variation/sleep disturbance; 1/5 about headache distribution; 2/5 about lifestyle factors (alcohol/drug use/smoking), 1/5 about computer/screen time; 1/5 about their last sight test; 1/5 about weight-loss; 1/5 about fever/intercurrent illnesses; 1/5 asked for more detailed PMH e.g. previous cancers; 1/5 asked about risk factors for carbon monoxide poisoning.

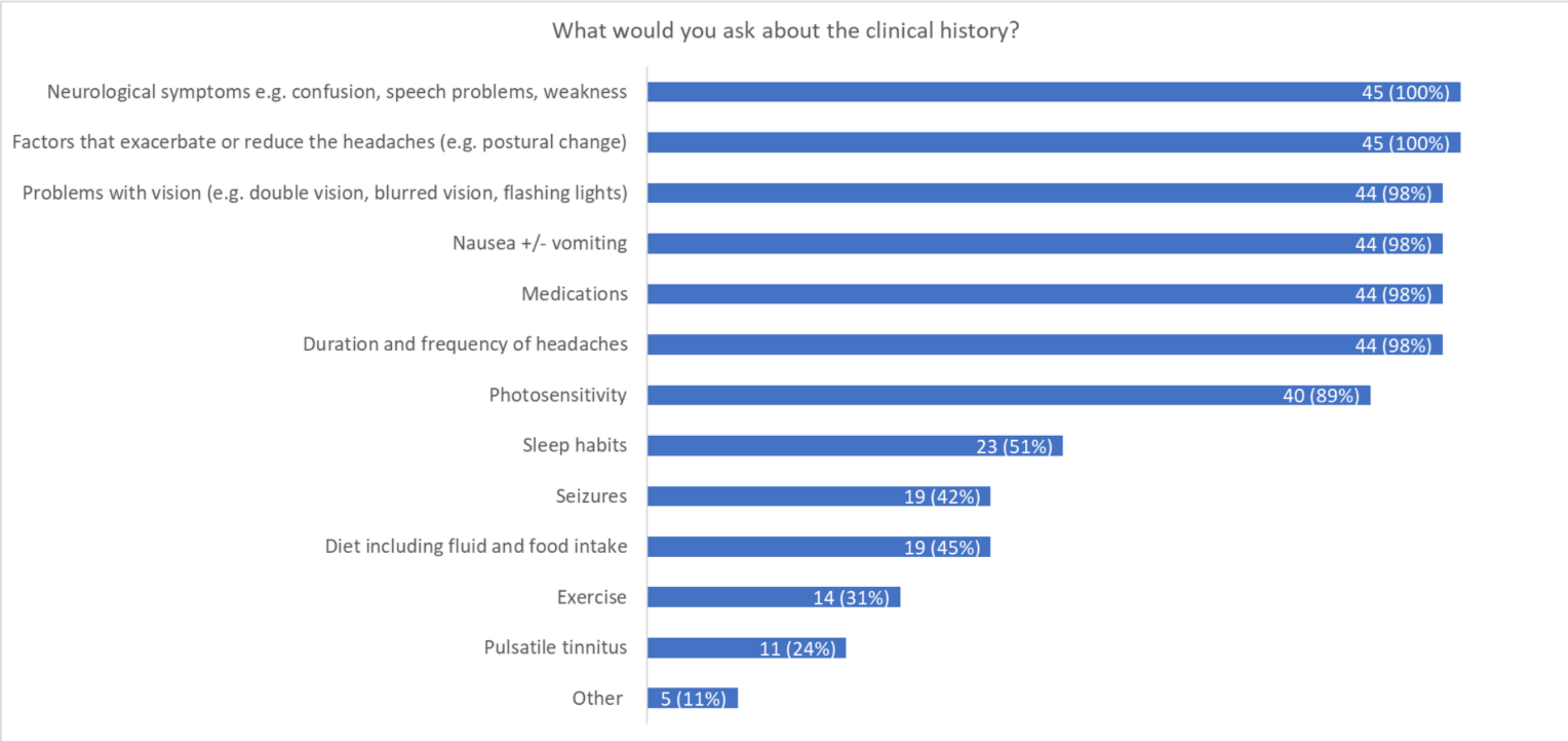

Figure 1: Further questions that GPs asked about case 1 in the clinical history.

<u>Q: For this patient with headaches, please select what you would routinely do next?</u>

Nearly all (43/45, 96%) GP respondents would next measure the blood pressure of case 1; 38/45 (84%) would examine the cranial nerves; and 23/45 (61%) would examine their arms and legs neurologically. Additional free text answers included: 4/45 (9%) fundoscopy (1/4 did not select they would examine the cranial nerves); 1/45, (2%) would “advise to get eye test or I would conduct fundoscopy” (but did not select they would examine the cranial nerves); 1/45 (2%) said “possibly none of the above if typical migraine”; 1/45 (2%) “Musculoskeletal, neck, check their medication e.g. contraceptive hormones” (Figure 2a).

Of the GPs who would conduct a cranial nerve exam, 37/38 (96%) would check pupil reflexes; 33/38 (87%) perform undilated direct ophthalmoscopy; 23/38 (61%) would assess visual fields to confrontation; and 11/38 (29%) would assess visual acuity (Figure 2b). Additionally, there were two GPs who said they would perform fundoscopy but did not consider this part of their cranial nerve examination.

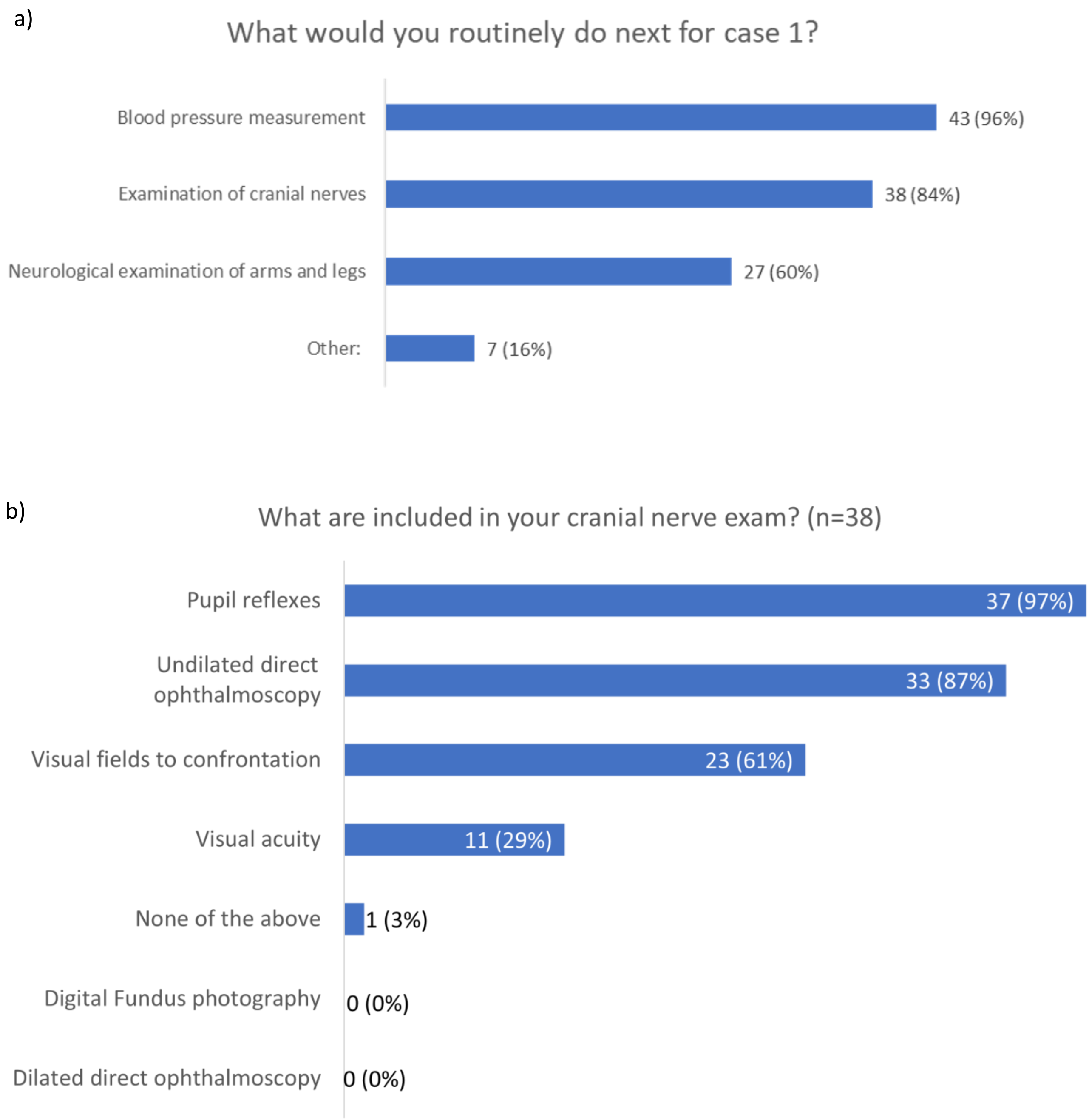

Figure 2: Most GPs would (a) measure BP and perform a cranial nerve examination for case 1, which (b) may include some vision tests.

<u>Q: Imagine you examine this patient's fundi with the direct ophthalmoscope and both optic discs look similar to the image above. Which of the following best describes this patient's optic disc appearances?</u>

The image shows a tilted and crowded disc. Only 3/45 (6.7%) GPs correctly identified the options 'optic disc drusen/pseudopapilloedema' (2/45) or 'crowded optic disc' (1/45). The majority (47%, 21/45) of GPs thought case vignette 1 had papilloedema and 14/45 (31%) stated they didn't know; 6/45 (13%) thought the discs appeared normal; 1/45 stated "uncertain if papilloedema"(Figure 3).

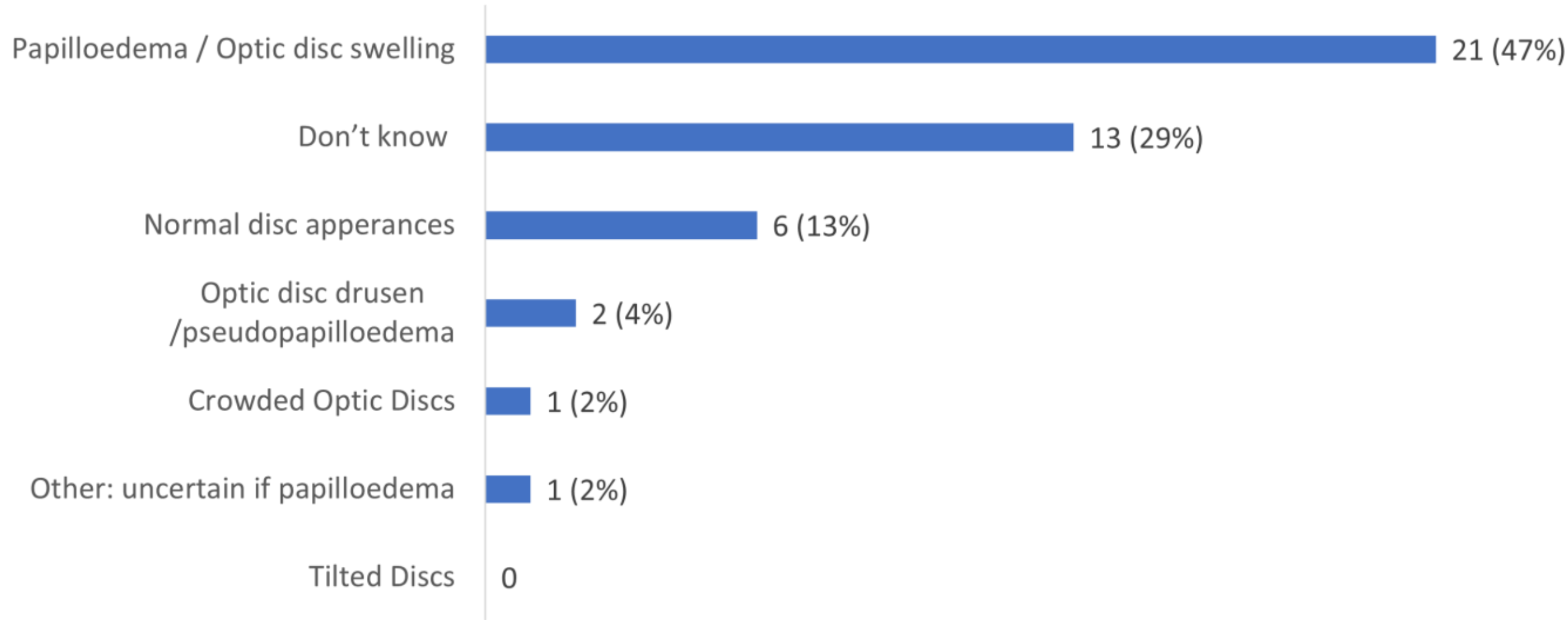

Figure 3: GPs had mixed opinions of the optic disc appearance of case vignette 1.

<u>Q: How confident do you feel in your answer to the previous question? Please rate your confidence on the scale of 1 to 5, where 1 means "not at all confident", and 5 means "very confident"</u>

GPs were not very confident in their assessment of the optic disc appearance (Figure 4).

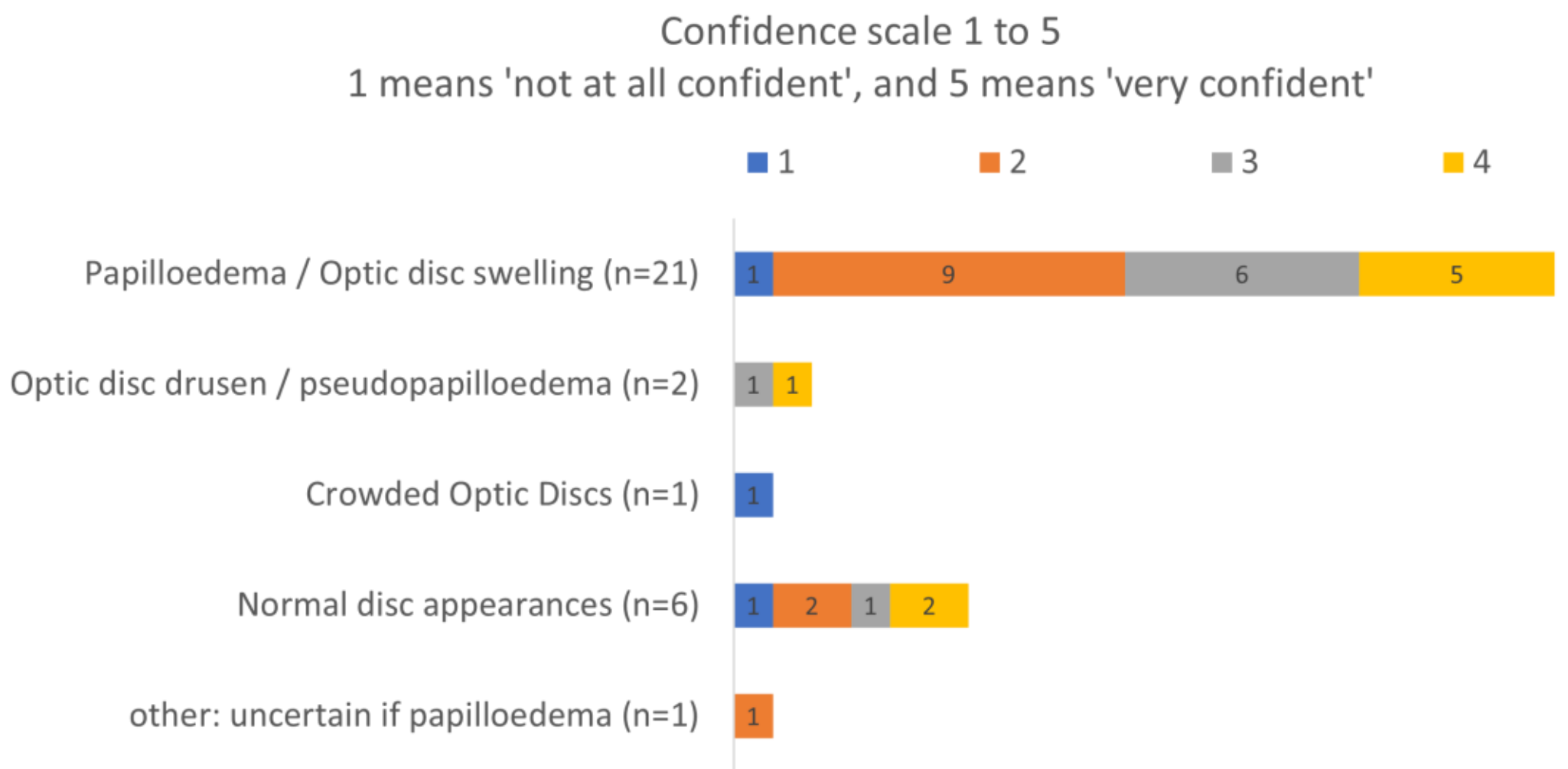

Figure 4: Few GPs were certain in their assessment of the optic disc appearance of case vignette 1.

An optional question invited GPs to describe any particular symptoms or signs that would alter their management of case 1. The most frequent answers were: neurological symptoms (14/40), vomiting/nausea (14/40), red flag headache features (e.g. waking from sleep, worse on leaning forward) (11/40), and visual disturbances (9/40). Other answers included: symptoms of raised ICP (4/40), severe hypertension/ high BP (3/40), seizures (3/40), abnormal examination findings (2/40), more unwell (2/40), photophobia (2/40), fever (1/40), lack of response to medication (1/40), occasional headaches (1/40).

B3 Case vignette 2: Additional results and figures

Case 2: You receive a referral letter from a local optometrist about a man in his thirties whom they have seen for a routine eye test. The patient went to see the optician because they thought their prescription needed to be updated, which it did. He is normally fit and healthy so no-one at the GP surgery has seen the patient for some time. He does not take medications. The optometrist has examined his fundi and says, in the referral letter, that the optic disc margins look raised, blurred, and indistinct.

GPs were invited to suggest any signs or symptoms that would influence their management of case 2. Responses included: red flag headache features (17/35) or red flags (3/35), neurological symptoms (16/35), visual disturbances (11/35), vomiting/nausea (6/35), high BP/severe hypertension (5/35), seizures (4/35), systemically unwell (4/35), abnormal examination findings (2/35), symptoms of raised ICP (1/35), loss of consciousness (1/35), symptoms of SOL (1/35). Three GPs said none.

Q: In situations when you are *UNCERTAIN the patient has optic disc swelling and you want to reassure the patient that they are getting the care they need, which three of the following do you think are of most benefit to the patient? Please rank your choices

Q: In situations when you are CERTAIN the patient has optic disc swelling and you want to reassure the patient that they are getting the care they need, which three of the following do you think are of most benefit to the patient? Please rank your choices

We developed a list of possible answers with our Patient and Public Involvement and Engagement group. We asked GPs to select the three best answers from this list when the diagnosis of papilloedema was certain /uncertain.

In both scenarios, most GPs thought patients would be most reassured by talking about what to expect following referral to hospital (37/45, 82%; 39/45, 87%), the possible range of differential diagnoses (36/45, 80%; 33/45, 73%) and waiting times for hospital appointments (20/45, 44%; 27/45, 60%). GPs appeared to think that information leaflets and online information were less useful (Figure 5).

One GP commented *"Standard practice is to give verbal explanation of possible diagnosis/safety netting which includes reasons to return and timeline and then back this up with written info, it isn't either/or and I don't think this question is phrased well. We now routinely text patients links rather than printing information (unless concerns re digital access).*

a)

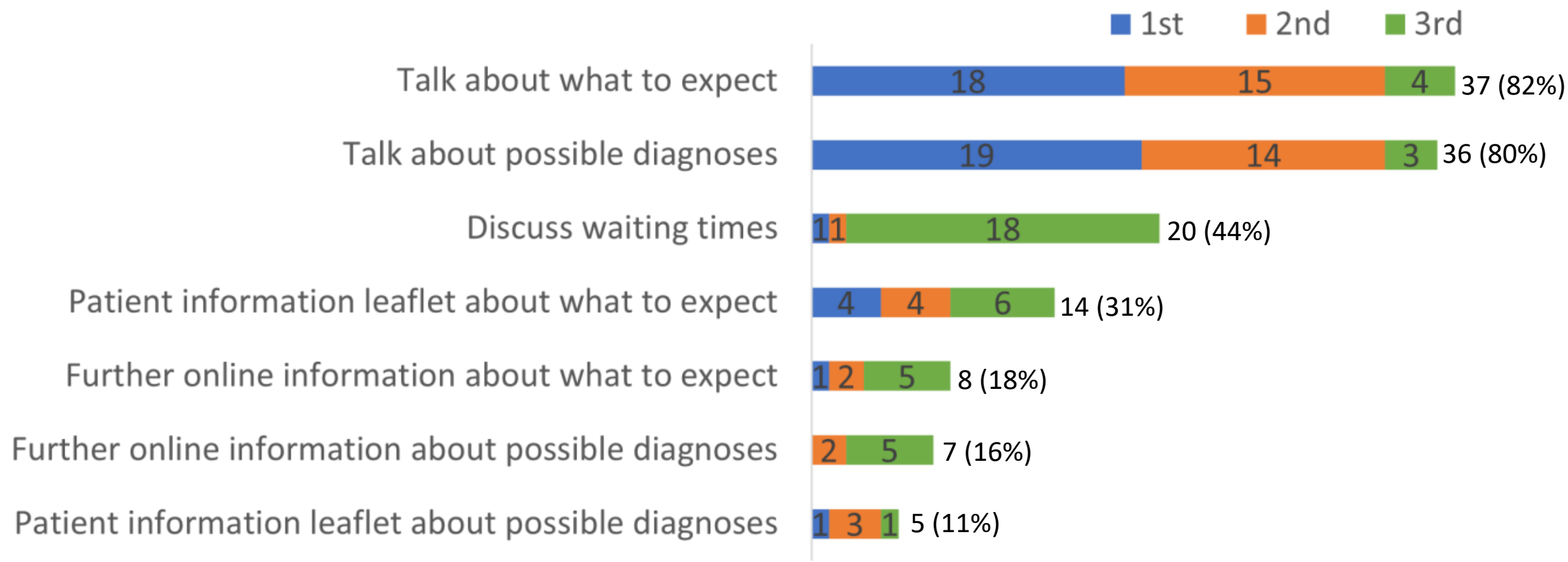

b)

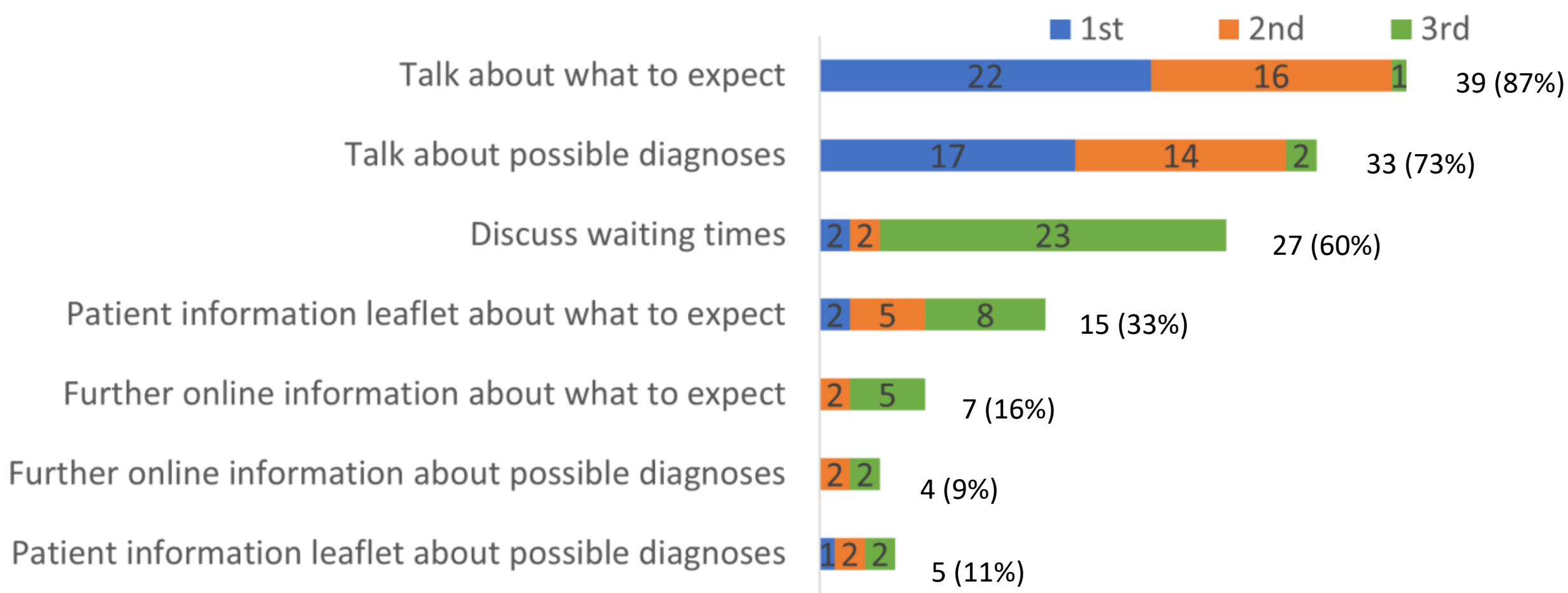

Figure 5: Most GPs preferred to verbally communicate information to patients about what to expect when referred to hospital and their differential diagnosis when they were (a) uncertain whether papilloedema was present and (b) certain the patient had papilloedema.

<u>Q: When you are certain the patient has optic disc swelling and you know that wait times for hospital appointments are long, how have you tried to speed up the referral?</u>

If concerned about waiting times for hospital appointments for a patient with definite papilloedema, most GPs responded they would emphasize red flags in their referral

letter/email (27/45, 60%); or call the ophthalmology department to discuss the patient (24/45, 53%). Around a third of GPs would recommend the patient is seen urgently/within a certain time-frame in the referral letter/email (15/45, 33%); or tell the patient to go to A&E (12/45, 27%). Free-text answers included: referral to eye casualty (1/45), 2WW neurology referral (1/45), and one GP commented *“we don't pick up optic disc swelling in general practice as we don't have the facilities for dilating pupils and proper photographic fundoscopy. So we don't do any of the above”*

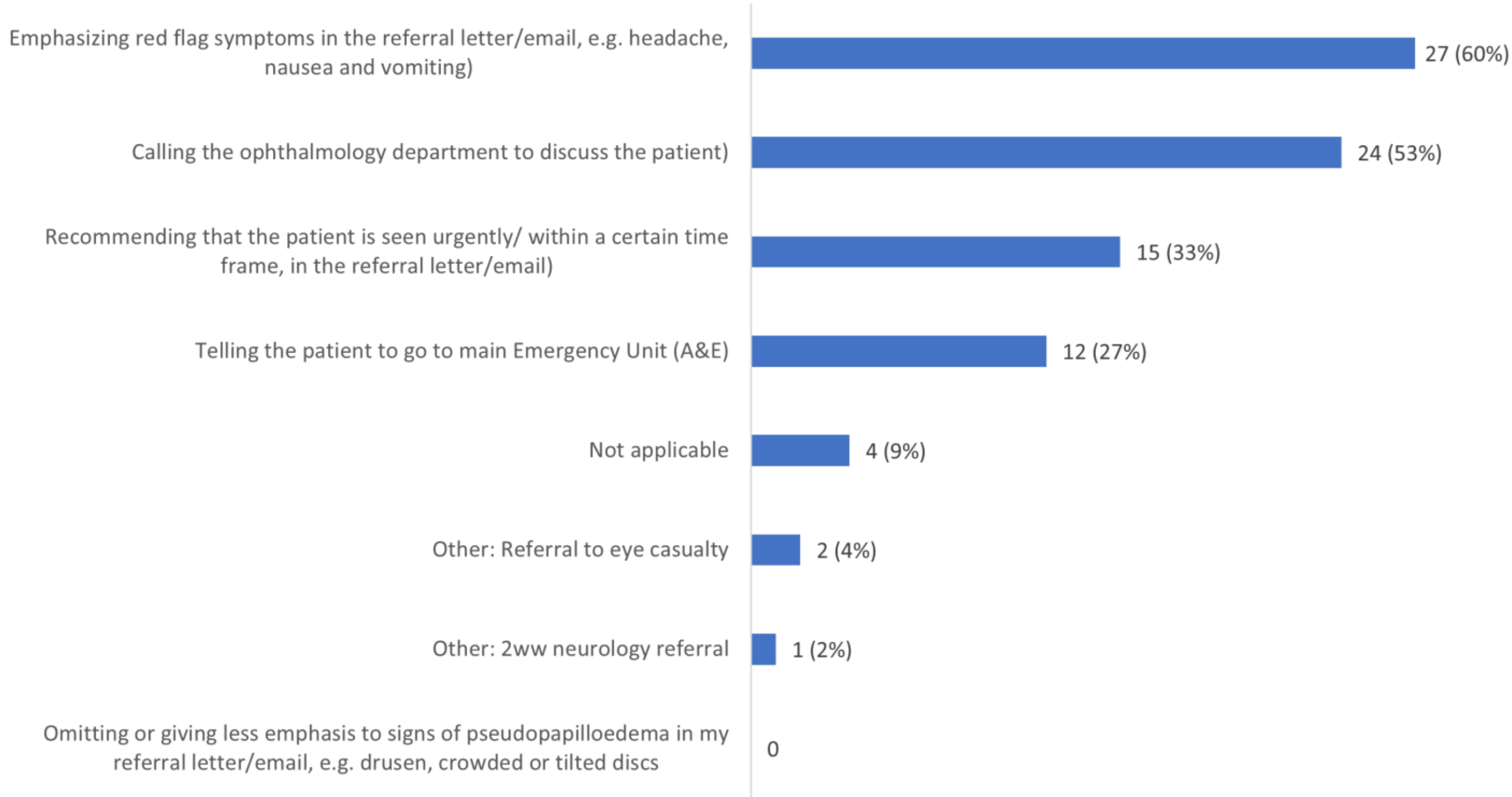

Figure 6: When certain that a patient has optic disc swelling and they are concerned about waiting times for hospital appointments, GPs will emphasize red flags symptoms or contact/send the patient to an emergency service.

<u>Q: In the last 5-10 years, do you think there have been any changes to the number of patients you have been referring to hospital with the following problems: Patients with headaches; Patients with headaches who you suspect to have papilloedema; Patients with headaches who the optometrist suspects has papilloedema; Patients who the optometrist suspects has papilloedema but are asymptomatic.</u>

Overall, most GPs thought referral trends had not changed over the last 5-10 years across the different patient scenarios, although nearly a quarter (9-11; 20-24%) stated that they did not know (Figure 7).

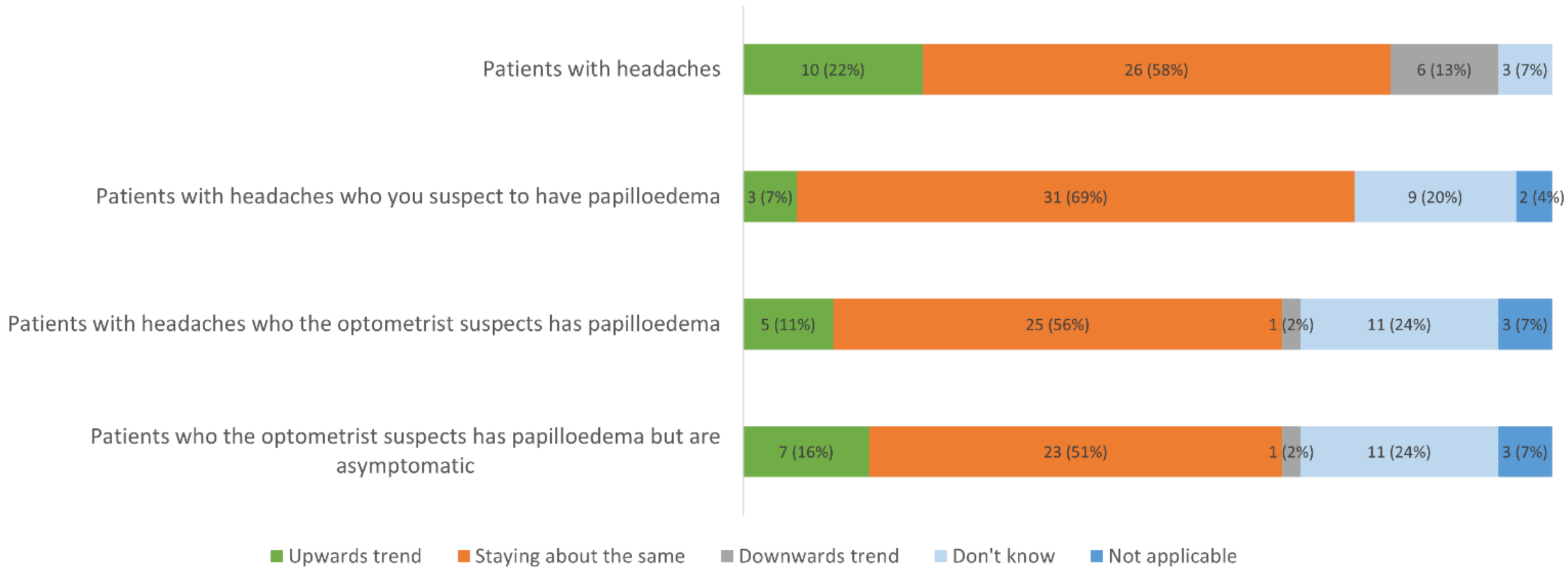

Figure 7: Most GPs thought referral trends had not changed over the last 5-10 years.

Q: Which one of the following events do you think has had the greatest impact on referrals for suspected papilloedema over the last 5-10 years? Answer options: Covid pandemic; Litigation case against an optometrist for missing papilloedema; or suggested alternative(s)

The majority (35/45) chose to not answer this question: 32/45 (71%) selected didn't know, and 3/45 (6.7%) selected not applicable. Those who selected Covid pandemic (3/45, 6.7%) thought fewer patients were seen during the pandemic that would present with suspected papilloedema; whereas those who chose the litigation case (5/45, 11%) thought fear/caution about the case would lead to defensive medicine. One GP's alternative suggestion was "*Guideline and case discussion with colleagues"*; and one GP stated "*I have referred very few, no influence"*

Q: Was there a difference between the number of adults and children you referred with possible optic disc swelling over the last year? (multiple choice)

The majority (18/45, 41%) of GPs did not answer, or stated they didn't know (8/45, 18%); however, 13/45 (30%) GPs said there was no difference, and 5 (11%) thought they had referred more adults than children.
