# Supplementary material for: Identification of papilloedema in general practice in England: a mixed-methods cross-sectional study
Source: BMJ Open. 2026 Oct 29;16(10):e121321. doi: 10.1136/bmjopen-2026-121321 (PMC13723832; doi:10.1136/bmjopen-2026-121321)
Supplement: Supplementary file 3 [file bmjopen-2026-121321.reviewer_comments.pdf]

PEER REVIEW HISTORY

BMJ Open publishes all reviews undertaken for accepted manuscripts. Reviewers are asked to complete a checklist review form and are provided with free text boxes to elaborate on their assessment. These free text comments are reproduced below.

ARTICLE DETAILS

Title (Provisional)

Identification of papilloedema in general practice in England: a mixed-methods cross-sectional study

Authors

Skrobot, Olivia; Chin, Jonathan A; Ridd, Matthew J; Merriel, Samuel W D; Atan, Denize; Huntley, Alyson

---

VERSION 1 - REVIEW

**Reviewer**	**1**
**Name**	**Lin, Haotian**
**Affiliation**	**State Key Laboratory of Ophthalmology, Zhongshan Ophthalmic Center, Sun Yat-sen University**
**Date**	**28-May-2026**
**COI**	**None**

Overall Evaluation: This study addresses the recognition and referral practices of papilloedema by primary care GPs, which is clinically and health-service relevant. Using a mixed-methods design combining surveys and semi-structured interviews, the study attempts to elucidate sources of misreferrals and diagnostic uncertainty. The combination of quantitative and qualitative data has the potential to provide useful insights into GP decision-making. However, there are several limitations regarding study design rigor, sample representativeness, integration of mixed methods, and depth of analysis. The manuscript would benefit from more clearer reporting, and stronger methodological transparency.

Major Comments:

1.Study Design: The study design should be described more accurately and cautiously. The authors describe this as a prospective study, but the study mainly consists of a cross-sectional survey with follow-up interviews. Authors should revise the description of the study design and use more precise terminology, which would help avoid overstating the methodological strength of the study.

2.Recruitment and Response Rate: The manuscript should provide clearer recruitment information and discuss potential selection bias more explicitly. However, it does not provide denominator data, response rates, or sufficient recruitment details. A participant flow diagram and regional distribution table would help readers assess representativeness and potential selection bias. Besides, statements may be overgeneralized given only 45 survey responses and 5 interviews and how the limited number of interviews may affect the depth and transferability of the qualitative findings.

3.Qualitative Data Sufficiency: The qualitative methodology requires additional justification and reporting detail. This manuscript does not clearly explain why five interviews were considered sufficient or whether thematic saturation/information power was achieved. The authors should briefly justify the interview sample size and discuss how regional variation may affect transferability of the findings.

4.Vignette and Image Validation: The development and validation process of the clinical vignettes and images should be described more clearly. The authors should clarify the source of the images, reasons for choosing these vignettes, expert review procedures, whether pilot testing was performed, and potential bias due to clinical clues in vignettes.

5.Reporting Transparency: The authors could consider supplying the manuscript with a COREQ checklist for interviews, a survey reporting guideline, and a GRAMMS statement for mixed methods. For instance, the manuscript should include interviewer background, coding processes, questionnaire development, and missing data handling .

Minor Comments:

1. Adjust abstract language from “robust and pragmatic” to “pragmatic”.

2. Standardize denominators for fundoscopy percentages.

3. Consider moving lengthy interview quotes from Table 1 to supplementary materials.

4. Clearly distinguish papilloedema, optic disc swelling, pseudopapilloedema, crowded disc, and optic disc drusen.

5. Address high number of “don’t know/not answered” in referral trends as uncertainty.

6. Add a paragraph on clinical and policy implications, distinguishing data-supported findings from hypotheses for further study.

**Reviewer**	**2**
**Name**	**Jalbert, Isabelle**
**Affiliation**	**The University of New South Wales, School of Optometry and Vision Science**
**Date**	**09-Jun-2026**

COI None

This is an interesting study that investigates an important question. My main concern with the manuscript relates to the very small number of participants involved. Because of that, it is not clear that the results provided are comprehensive or truly representative of the GP perspectives on identifying papilloedema. A clear sample size justification is required. It is uncertain whether the quantitative (n=45) or qualitative (n=5) aspects of the study had adequate sample. This is a serious limitation for this manuscript. An explanation for why only six of 15 NIHR clinical Research Networks participated in the study is also required.

Participants were asked whether they "thought hospital referrals from GPs and optometrists had changed over the last 5-10 years for suspected papilloedema" (page 13). The question in Appendix B3 refers to a litigation case against an optometrist for missing papilloedema. It thus appears that there is some important relevant context that was not included in the introduction and may not be understood by non UK readers or those from non eyecare professions. This context and how the investigators feels that hospital referrals have changed should be introduced and explained in the introduction.

A strength of the study was the inclusion of a step involving consultation with Public and Patient Involvement and Engagement (PPIE) groups. A nice description of how the thematic analysis of the qualitative data was conducted is provided. The description of the methods could include more details at other times:

- Briefly describe how the questionnaire was designed. Was the content based on an existing questionnaire? Was a literature review conducted? Was the questionnaire piloted? Was it validated?

- Briefly describe how the "bespoke" topic guide was developed for the semi-structured interviews. What this based on existing literature or expert opinion or something else?

Define GP and PPIE (page 7) for non UK readers.

VERSION 1 - AUTHOR RESPONSE

Reviewer: 1
Dr. Haotian Lin, State Key Laboratory of Ophthalmology, Zhongshan Ophthalmic

Major comments
1. Study Design: The study design should be described more accurately and cautiously.

The authors describe this as a prospective study, but the study mainly consists of a

cross-sectional survey with follow-up interviews. Authors should revise the description

of the study design and use more precise terminology, which would help avoid overstating the methodological strength of the study.

As cited above we have edited the title to:

"Identification of papilloedema in general practice in England: a mixed-methods cross-sectional study"

And the study design under abstract and methods is:

"A mixed-methods cross-sectional study of GPs in England."

2. Recruitment and Response Rate: The manuscript should provide clearer recruitment information and discuss potential selection bias more explicitly. However, it does not provide denominator data, response rates, or sufficient recruitment details. A participant flow diagram and regional distribution table would help readers assess representativeness and potential selection bias. Besides, statements may be overgeneralized given only 45 survey responses and 5 interviews and how the limited number of interviews may affect the depth and transferability of the qualitative findings.

We have designed our study with a mixed method approach as described in Huntley et al, *BMJ Open* 2024 (doi:10.1136/ bmjopen-2024-090521). Because the number of recipients of the study invitations was not recorded, we have acknowledged that our methods do not allow us to determine a response rate. We are not convinced of the value of a flow diagram as the study is not configured in that way - people either responded or didn't respond on seeing our written invitations as participation relied on GPs to volunteer themselves to take part. We cannot estimate how many saw the study invitations, wanted to respond but did not/could not, or were not interested. We state the 6 NIHR clinical research networks from where participants were recruited from in the methods.

We appreciate the relatively low numbers of participants, particularly in the interviews. Interviewees were purposively selected from different areas of England among the pool of GPs who had completed the survey and the optional consent to be contacted for interview. The aim of our survey and interviews was to capture what is currently happening in practice, and any variation within that, in greater depth. We were not aiming for transferability/generalisability (applying found information on people we did

not interview), but were aiming for a greater depth in understanding the context and influencing factors that came up in the survey. Our aims were very much based in fact-finding and understanding the clinical context more thoroughly. We feel we have acknowledged these limitations in the relevant sections of the manuscript.

We have adapted the bullet point in the Strengths and Limitations after the abstract to read:

"Whilst 6 clinical research networks in England agreed to disseminate the invitation to practicing GPs, these networks did not cover the North of England."

We have adapted the paragraph in the Strengths and Limitations section of the discussion to read: *CRNs were an effective means to disseminate our questionnaire to GPs; however, none participated from the north of England and therefore recruitment from general practice from this region was not possible. The sample of GPs who took part was small and could be open to bias. Considering the known variation in referral pathways available to GPs across the nation and that respondents were member GP surgeries, limit the generalisability of the findings. However, we feel confident that the interviewees were representative of the survey respondents and more interviews would have given diminishing returns.*

3.Qualitative Data Sufficiency: The qualitative methodology requires additional justification and reporting detail. This manuscript does not clearly explain why five interviews were considered sufficient or whether thematic saturation/information power was achieved. The authors should briefly justify the interview sample size and discuss how regional variation may affect transferability of the findings.

As mentioned above, the purpose of our semi-structured interviews was to add real life experience and context to the semi-quantitative data from the surveys. Hence this was not a qualitative study but a mixed methods study that used qualitative analysis methods to analyse the content of the semi-structured interviews.

We feel that our 5 interviews were representative of our 45 questionnaire respondents. Our study follows the thinking of Saunders *et al* on data saturation (doi:10.1007/s11135-017-0574-8) and Malterud and colleagues on information power (doi: 10.1177/1049732315617444); both manuscripts suggest that the optimal size of a sample relies on factors such as the aim of the study, quality of content, and the analysis strategy. In this study, we feel confident that more interviews would have given diminishing returns.

In the strengths and limitations section of the original manuscript on p24-25, we had already stated:

"*Participant recruitment from general practice was low in some regions, e.g., no-one participated from the north of England. These issues limit the generalisability of the findings.*"

In response to the reviewer, we have further added this paragraph:

"CRNs were an effective means to disseminate our questionnaire to GPs; however, none participated from the north of England and therefore recruitment from general practice from this region was not possible. The sample of GPs who took part was small and could be open to bias. Considering the known variation in referral pathways available to GPs across the nation and that respondents were member GP surgeries, limit the generalisability of the findings. However, we feel confident that the interviewees were representative of the survey respondents and more interviews would have given diminishing returns."

4.Vignette and Image Validation: The development and validation process of the clinical vignettes and images should be described more clearly. The authors should clarify the source of the images, reasons for choosing these vignettes, expert review procedures, whether pilot testing was performed, and potential bias due to clinical clues in vignettes.

The two case vignettes were based on the real-world presentations of patients referred to the neuro-ophthalmology service of one of the co-authors (DA) from the community. The clinical scenarios are two examples that appear to create the greatest diagnostic uncertainty, as reported in McNicholl *et al* (doi: 10.1038/s41433-022-02310-0). In this paper, we show most false positive referrals for suspected papilloedema are people with benign headache symptoms or they have uncertain disc appearances that could be interpreted as pseudopapilloedema or papilloedema (with blurred or elevated or indistinct margins). Hence the case vignettes were designed to reflect these two common clinical scenarios. The fundal image for case 1 was used with the consent of the patient.

As part of the larger study described in our protocol paper (doi:10.1136/ bmjopen-2024-090521), the survey questions were written and piloted with the help of optometrists, ophthalmologists, emergency care clinicians and neurologists, as well as GPs, before they were disseminated to these professions via the appropriate channels. The first case complained of headache and had a photo of pseudopapilloedema; the second case was an optometrist referral letter describing an asymptomatic patient with 'raised, blurred and indistinct optic disc margins'. We consider these 'clues' in the case vignettes to be representative of the typical presentations of patients referred to hospital for suspected papilloedema rather than a source of 'bias'. It is normal clinical practice for health professionals to assess patients based on their clinical presentations and we wanted to replicate this in the surveys.

We have added further detail on the points described above to the methods section on page 8 under the subheading “Questionnaire”.

5.Reporting Transparency: The authors could consider supplying the manuscript with a COREQ checklist for interviews, a survey reporting guideline, and a GRAMMS statement for mixed methods. For instance, the manuscript should include interviewer background, coding processes, questionnaire development, and missing data handling.

We have followed the editor’s suggestion of using the STROBE reporting checklist for cross-sectional studies, which captures the broad methodological approach we took to obtain our data. We feel this is more appropriate that the COREQ checklist which is designed for qualitative studies. We agree that addressing the GRAMMS criteria improves the transparency of the mixed method approach we have taken in our research and have included the GRAMMS criteria alongside the STROBE checklist in the supplementary material. We have also referred to these reporting checklists in the methods section of the manuscript, under the heading “Reporting”. We have reaffirmed our approach with brief statements where appropriate in the results. We have included more detail about questionnaire development in the questionnaire section of the methods and missing data handling in analysis section of the methods and results under “participants”.

Minor Comments:
Adjust abstract language from “robust and pragmatic” to “pragmatic”.

We have removed this bullet point.
Standardize denominators for fundoscopy percentages.

We have done this
Consider moving lengthy interview quotes from Table 1 to supplementary materials.

We would prefer to keep the interview quotes in the main paper but will defer to the preferred style of the journal.
Clearly distinguish papilloedema, optic disc swelling, pseudopapilloedema, crowded disc, and optic disc drusen.

We have added an extra table. Table 1 is now a glossary of terms and appears after the introduction and before the methods. Table 1 (quotes) has been relabelled as table 2.

Address high number of “don’t know/not answered” in referral trends as uncertainty.

We have added this to a sentence in the discussion: *Despite the rise in referrals for suspected papilloedema reported by hospital eye services in the UK, most GPs hadn’t appreciated any changes in referral rates from optometrists over the last 5-10 years, although some said optometrists could refer patients directly to hospital in their area*

and nearly a quarter were uncertain about referral trends.

Add a paragraph on clinical and policy implications, distinguishing data-supported findings from hypotheses for further study.

We have rephrased the discussion on page 24 to say:

"GPs in our study expressed a need for clearer guidance on the appropriate use of community optometry services and referral pathways to secondary care for patients presenting with headache and suspected papilloedema. Such guidance may support more consistent decision-making, facilitate timely identification of patients with raised ICP, and optimise the use of healthcare resources. However, further research is required to evaluate the clinical effectiveness and cost-effectiveness of implementing these pathways, including their impact on diagnostic timeliness, patient outcomes, referral patterns, and NHS resource utilisation."

Similarly, the conclusions to the manuscript have been revised to say:

"GPs reported limited exposure to, and confidence in identifying headache patients with papilloedema from raised ICP. Participants highlighted the need for additional training, and sufficient consultation time to assess for papilloedema, an important headache red flag. GPs supported the development of clear clinical guidance to aid: 1. Recognition of headache presentations and signs of papilloedema from raised ICP requiring urgent hospital referral; 2. appropriate referral to community optometrists and interpretation of ophthalmic findings; and 3. management of patients with benign headache disorders or pseudopapilloedema within the community. Further research is needed to determine whether implementation of such guidance could reduce diagnostic delays for patients with headache and papilloedema due to raised ICP, while improving referral pathways and reducing unnecessary healthcare utilisation and costs."

These edits to the manuscript clearly describe clinical and policy implications, and distinguish data supported findings from hypotheses to be tested in further study.

Reviewer: 2
Prof. Isabelle Jalbert, The University of New South Wales

1.This is an interesting study that investigates an important question. My main concern with the manuscript relates to the very small number of participants involved. Because of that, it is not clear that the results provided are comprehensive or truly representative of the GP perspectives on identifying papilloedema. A clear sample size justification is required. It is uncertain whether the quantitative (n=45) or qualitative

(n=5) aspects of the study had adequate sample. This is a serious limitation for this manuscript. An explanation for why only six of 15 NIHR clinical Research Networks participated in the study is also required.

We have addressed our sample size across our two methods in response to reviewer one's comments

2.Participants were asked whether they "thought hospital referrals from GPs and optometrists had changed over the last 5-10 years for suspected papilloedema" (page 13). The question in Appendix B3 refers to a litigation case against an optometrist for missing papilloedema. It thus appears that there is some important relevant context that was not included in the introduction and may not be understood by non UK readers or those from non eyecare professions. This context and how the investigators feels that hospital referrals have changed should be introduced and explained in the introduction.

We have added to the introduction:

"*The conviction of an optometrist in England for gross negligence manslaughter after they failed to identify papilloedema in a child who later died has made optometrists more anxious about missing the diagnosis*. As a result, hospital eye services across the UK have reported large numbers of optometrist referrals of people with suspected papilloedema…"

3.A strength of the study was the inclusion of a step involving consultation with Public and Patient Involvement and Engagement (PPIE) groups. A nice description of how the thematic analysis of the qualitative data was conducted is provided. The description of the methods could include more details at other times: Briefly describe how the questionnaire was designed. Was the content based on an existing questionnaire? Was a literature review conducted? Was the questionnaire piloted? Was it validated?

We have included the sentence in the methods:

"*The study team, including two academic GPs (MJR, SWDM), a neuro-ophthalmologist (DA) and researchers with expertise in mixed methods and evidence synthesis (OS, AH), contributed their respective subject knowledge to write and pilot the questions*."

Additionally, we have added:

"The case vignettes were based on the clinical presentations of real-world patients referred to a UK-based hospital eye service (DA), which typically create the greatest diagnostic uncertainty."

Briefly describe how the "bespoke" topic guide was developed for the semi-structured interviews. What this based on existing literature or expert opinion or something else?

We have added to the end of the sentence referring to a "bespoke topic guide":

“…, designed by the study team to provide context to the semi-quantitative data of the survey by exploring real life experiences.”

Define GP and PPIE (page 7) for non-UK readers.

We have defined these.

VERSION 2 - REVIEW

Reviewer **2**

Name **Jalbert, Isabelle**

Affiliation **The University of New South Wales, School of Optometry and Vision Science**

Date **23-Aug-2026**

COI

Thank you for the considered revision to this manuscript. I am comfortable that the reviewers' requests have mostly been adequately addressed. The following queries remain:

- The authors' response with regard to the low sample size is inadequate and the changes to the manuscript to not appropriately address the significant risk of bias. This should be further revised to strengthen the statements regarding the high risk of sample bias and sampling error.

- In the methods on page 10 in the section on Reporting, I am comfortable with the decision to include both the STROBE and GRAMM checklists, however, could the authors cite the relevant papers that describe these checklists in this section.

- I am supportive of keeping the interview quotes in the main paper.

- Provide a supporting reference (citation) to the statement introducing the gross negligence manslaughter optometrist case.

- Provide more details on the questionnaire design and validation. How was it piloted, confirm that it was piloted on optometrists that were not part of the research team, stated how many participants were involved in the pilot and what changes were made as a result of piloting.

VERSION 2 - AUTHOR RESPONSE

Reviewer: 2

Prof. Isabelle Jalbert, The University of New South Wales

Comments to the Author:

Thank you for the considered revision to this manuscript. I am comfortable that the reviewers' requests have mostly been adequately addressed. The following queries remain:

Comment 1

The authors' response with regard to the low sample size is inadequate and the changes to the manuscript to not appropriately address the significant risk of bias. This should be further revised to strengthen the statements regarding the high risk of sample bias and sampling error.

Response

We have added/edited to the text in the discussion, emphasising the potential bias and how it may have occurred. We have also given extra information about the greater DIPP project to further contextualise our findings.

‘We acknowledge that the number of GPs participating in the survey and interviews was modest and limited to members of the CRN. As referral pathways for suspected papilloedema vary across the UK, it is possible that some perspectives were not captured, which may limit the generalisability of the findings.

However, this work should be considered within the broader context of the DIPP study, which also included surveys and interviews with optometrists and ophthalmologists regarding the same referral pathway.15 Although these findings have been reported separately for practical reasons, participants from different professional groups frequently commented on the roles, experiences, and challenges faced by others within the pathway. The consistency of these observations with the views expressed by GPs provides reassurance that no major sources of bias were evident. In addition, the interview data were broadly consistent with the survey findings and provided valuable contextual insights that helped explain and confirm the quantitative results. Taken together, we believe that the participants provided a sufficiently diverse range of perspectives to address the study objectives. While a larger survey and additional interviews may have identified further nuances, we consider it unlikely that they would have materially altered the principal findings, particularly given the practical constraints of finite research resources. We also added a point in the strengths and limitations to cover this.’

- *The survey and interview sample sizes were modest; however, within the context of the broader study, there were no obvious indications of bias.*

Comment 2

In the methods on page 10 in the section on Reporting, I am comfortable with the decision to include both the STROBE and GRAMM checklists, however, could the authors cite the relevant papers that describe these checklists in this section.

Response

Thank you, these have been added in

Comment 3

I am supportive of keeping the interview quotes in the main paper.

Response

Thank you

Comment 4

Provide a supporting reference (citation) to the statement introducing the gross negligence manslaughter optometrist case.

Response

We have now cited this referenced appropriately

Comment 5

Provide more details on the questionnaire design and validation. How was it piloted, confirm that it was piloted on optometrists that were not part of the research team, stated how many participants were involved in the pilot and what changes were made as a result of piloting.

Response

We have added this extra text in the paper.

'The DIPP surveys were initially developed by the neuro-ophthalmologist (DA) and health services researchers (ALH, OAS) within the DIPP study team. The surveys were subsequently tailored to each professional group within the referral pathway following consultation with representatives from the relevant disciplines. Feedback was obtained through meetings and email correspondence with at least one health professional from each target group, including general practitioners, optometrists, emergency care clinicians, ophthalmologists and neurologists.

The GP survey was piloted by MJR and SWDM, who were invited to join the DIPP study team specifically to provide general practice perspectives and expertise. Following pilot testing, several modifications were made to improve the clarity, relevance, and accessibility of the survey for GPs. For example, the question in Case 1 asking, "What

would you ask about in the clinical history?" was changed from free text response to a multiple-choice format. In addition, a named legal case included in a question exploring factors influencing referral decisions was replaced with the more general description, "Litigation case against an optometrist for missing papilloedema"

Reviewer: 2

No action needed
